# Consumer Views on Health Applications of Consumer Digital Data and Health Privacy Among US Adults: Qualitative Interview Study

**DOI:** 10.2196/29395

**Published:** 2021-06-09

**Authors:** David Grande, Xochitl Luna Marti, Raina M Merchant, David A Asch, Abby Dolan, Meghana Sharma, Carolyn C Cannuscio

**Affiliations:** 1 Division of General Internal Medicine University of Pennsylvania Philadelphia, PA United States; 2 Leonard Davis Institute of Health Economics University of Pennsylvania Philadelphia, PA United States; 3 Department of Emergency Medicine University of Pennsylvania Philadelphia, PA United States; 4 Department of Family Medicine and Community Health University of Pennsylvania Philadelphia, PA United States

**Keywords:** health privacy, digital health privacy, privacy law, health law, digital epidemiology

## Abstract

**Background:**

In 2020, the number of internet users surpassed 4.6 billion. Individuals who create and share digital data can leave a trail of information about their habits and preferences that collectively generate a digital footprint. Studies have shown that digital footprints can reveal important information regarding an individual’s health status, ranging from diet and exercise to depression. Uses of digital applications have accelerated during the COVID-19 pandemic where public health organizations have utilized technology to reduce the burden of transmission, ultimately leading to policy discussions about digital health privacy. Though US consumers report feeling concerned about the way their personal data is used, they continue to use digital technologies.

**Objective:**

This study aimed to understand the extent to which consumers recognize possible health applications of their digital data and identify their most salient concerns around digital health privacy.

**Methods:**

We conducted semistructured interviews with a diverse national sample of US adults from November 2018 to January 2019. Participants were recruited from the Ipsos KnowledgePanel, a nationally representative panel. Participants were asked to reflect on their own use of digital technology, rate various sources of digital information, and consider several hypothetical scenarios with varying sources and health-related applications of personal digital information.

**Results:**

The final cohort included a diverse national sample of 45 US consumers. Participants were generally unaware what consumer digital data might reveal about their health. They also revealed limited knowledge of current data collection and aggregation practices. When responding to specific scenarios with health-related applications of data, they had difficulty weighing the benefits and harms but expressed a desire for privacy protection. They saw benefits in using digital data to improve health, but wanted limits to health programs’ use of consumer digital data.

**Conclusions:**

Current privacy restrictions on health-related data are premised on the notion that these data are derived only from medical encounters. Given that an increasing amount of health-related data is derived from digital footprints in consumer settings, our findings suggest the need for greater transparency of data collection and uses, and broader health privacy protections.

## Introduction

In 2020, internet users spent 1.25 billion years online. The average internet user spends 6 hours and 43 minutes online each day [1], leaving a trail of information about his/her habits and preferences that collectively generates a digital footprint.

A consumer’s digital footprint can reveal health-related behaviors, such as diet and physical activity, that can predict health [2]. For example, data from social media sites, including Twitter and Facebook, can be used to screen for signs of depression, suicidal ideation, and sleep disorders based on user activity and language patterns [3-6]. These applications of data toward health purposes erase past distinctions between health and nonhealth data [7,8].

The COVID-19 pandemic has accelerated these applications. Smartphone location services and cashless transactions have been used to identify and track COVID-19–positive individuals [9]. Social media posts have been used to track reports of physical symptoms of COVID-19 and mental health sequelae of the pandemic [10,11]. Apple and Google have updated their smartphone operating systems to enable tracking of human-to-human interactions to enable digital contact tracing [12]. These public health applications of consumer digital data have triggered global debates and urgent policy discussions about digital health privacy [13].

A majority of Americans simultaneously reported feeling concerned about how their personal data are being used and yet continue to use digital technologies. Many believe it is impossible to go through daily life without having personal data collected by companies or the government, with 80% feeling they lack control over what data are collected and 63% admitting having little to no understanding about data privacy laws and regulations [14].

We interviewed a diverse sample of American consumers to evaluate their awareness of the health applications of their digital data and identify their privacy views and concerns.

## Methods

### Participants

Interviews were conducted between November 1, 2018, and January 31, 2019. Participants were identified from the web-enabled Ipsos KnowledgePanel, a probability-based panel designed to be representative of the US population [15]. For this study, Ipsos provided the contact information of 200 participants drawn from the nationally representative panel with even distribution for various demographic variables (gender, race, age, household income, and geographic region) to ensure diversity in the study sample. From these, team members (XLM and AL) completed 45 interviews, ensuring equal proportions of participants were included from each category so that the sample of 45 reflected the larger sample of 200. Participants who completed the interview were paid US $50.

This study protocol was reviewed by the Institutional Review Board of the University of Pennsylvania and declared exempt. Verbal consent was obtained from participants prior to their interview. All results are reported following the Standards for Reporting Qualitative Research (SRQR) reporting guidelines.

### Design

Telephone interviews were conducted using a semistructured qualitative interview guide (Multimedia Appendix 1). This guide was informed by a consequential ethics framework, in which the presence or absence of a substantial risk of harm from a loss of privacy determines the need for protections [16]. The guide asked consumers to reflect on their own use of digital technology, evaluate sources of digital information, and consider several hypothetical scenarios with varying uses of digital information (scenarios shown in Table 1). While the scenarios were hypothetical, they were developed based on plausible use cases in today’s landscape of consumer digital data. The interviews lasted 30 to 45 minutes, and were conducted by research coordinators (AL and XLM) trained in qualitative interviewing methods. The audio recordings were sent to a professional transcription service (ADA Transcription) where they were deidentified. Transcripts were uploaded to NVivo Version 12 for data analysis [17].

**Table 1:** Scenarios.

No.	Text	Source	User	Use
1	*Your health insurance company is trying to find ways to keep people healthier and save money. They have found that consumers that buy certain kinds of food are more likely to develop diabetes. The insurance company is planning a program where they will access the grocery shopping records of their patients from grocery stores. The health insurance company will use this information to find out who is at high risk of developing diabetes, then send those people tips and advice on how they can prevent diabetes by making changes to the food they buy.*	Grocery shopping records	Health insurance company	Prevent diabetes
2	*Your doctor’s office is trying to find ways to prevent people from getting sick and needing to go to the hospital. They have found that patients that search on the internet for certain symptoms are more likely to get sick and need to go to an emergency room. Your doctor’s office is planning a new program where they will access internet searches of their patients and contact patients that search for certain symptoms to try to start treatment sooner.*	Internet search history	Doctor’s office	Diagnose disease earlier
3	*University researchers are trying to find ways to prevent cancer. Researchers at a nearby university hospital are starting a research study where they will track patients over time to try to determine causes of cancer. In addition to using medical records, the research team will use location information from patient’s smartphones so they can study how the places where people spend most of their time impact their risk of getting cancer. The researchers want to use this knowledge to help develop public health strategies in the future that could reduce the number of people with cancer.*	Electronic health record and location data from a smartphone	University researchers	Research to identify risk factors for cancer
4	*DigiHealth is a company selling a new smartphone app that can automatically collect and store information on places users visit and the food they eat so that it can give advice on ways to lower their risk of obesity. The app tracks where users go using location services on their smartphone and tracks what they eat by having them upload a picture of their meals. DigiHealth is able to offer the app for free because it shares user information with advertisers so they can send out grocery coupons.*	Location data and photos from a smartphone	Technology company	Send grocery coupons for healthier foods to prevent obesity

### Analysis

The study team developed a codebook through line-by-line iterative reading and notation of transcripts, which produced 12 key codes [18]. Some codes were used a priori in order to compare data from consumers to the data from similar interviews conducted with experts [7]. Two research coordinators used the codebook to complete coding (AL and XLM). In order to establish agreement, five interview transcripts were double coded. Interrater reliability was high, with overall agreement (97.5%) and Cohen kappa of 0.7. After agreement was established, the remaining transcripts were coded individually. Interviews were coded sequentially in the order they were conducted. A memo was written, and it thematically summarized the content of each code. These memos were reviewed collectively by the study team in order to identify salient findings. The study team found that they were no longer learning new information in the process of coding new transcripts. Therefore, through coding and analysis meetings, key study personnel reached a consensus that thematic saturation was reached. The results from these interviews are reported along with supporting quotes to highlight relevant findings.

## Results

### Participants

The sampling methods produced a final cohort of 45 consumers with diversity in gender, race, age, household income, and geographical distribution (Table 2).

**Table 2:** Demographics of the participants (N=45).

Characteristic		Value, n (%)
**Gender**		
	Male	24 (53%)
	Female	21 (47%)
**Race**		
	Minority (non-White)	22 (49%)
	White/Caucasian	23 (51%)
**Age (years)**		
	18-30	14 (31%)
	31-45	10 (22%)
	46-60	11 (24%)
	>60	10 (22%)
**Household income (US$)**		
	≤24,999	10 (22%)
	25,000-49,999	11 (24%)
	50,000-99,999	13 (29%)
	≥100,000	11 (24%)
**Region**		
	Midwest	12 (27%)
	Northeast	10 (22%)
	South	13 (29%)
	West	10 (22%)
**Education**		
	Less than high school	3 (6%)
	High school	12 (27%)
	Some college	13 (29%)
	Bachelor’s degree or higher	17 (38%)

### Part 1: Views on Digital Privacy and Health

The first portion of participant interviews focused on consumers’ understanding of current data use as well as their views on digital health privacy. Key themes are described below, and illustrative quotes are presented in Table 3.

**Table 3:** Key themes from consumer interviews.

Theme	Illustrative quote
Lack of consumer understanding	*I don’t really have a great knowledge of what they gather from me. I know when I signed up and you gotta go through the cursory that says do you agree to all this, there’s just pages of stuff and you just say, like everything else, you just say yeah, I agree.* *I don’t think Google collects any information about me when I use it.* *My health? I mean, they wouldn’t think I have any health issue...I don’t make any payments in regards to my health* *.* *I don’t think they could learn nothing. You don’t put your health information on there. Like what would they know from Instagram?* *I don’t necessarily know what they would gain about my health overall other than sometimes when I’m sick, I’ll research my symptoms online or something like that.*
Benefits	*Well, I think the biggest benefit that I can think of –the reason that I use Reddit is because it’s beneficial to feel like I belong to a handful of communities. And so, I think I deepen my interest –the things that I’m interested in feel more meaningful when I read what other people are talking about.* *I think it would help them –if they were gathering that information, they would use it for marketing and potential sales, how they go about structuring better ways to advertise to persons in organizations.* *Well, benefits –I get to find out things that my friends are doing that they want me to know, because otherwise they wouldn’t post it. But it’s also a double-edged sword in that –basically, that –that those –whatever they’re talking about –whatever –however I’m reacting to it, is more information for advertisers to target me with.*
Harms	*Well, I think that information should just be between me and my doctor. I wouldn’t want any advertisers to be able to learn that information, and I wouldn’t want anything that I wouldn’t share with my family or friends to come out in the public regarding anything that was physically going on with me.* *Well, I think the –some of the worry is that the Fitbit–I don’t have one or use one, but –so the worry is that it might provide inaccurate data that might be used against me. For example, if I applied for life insurance, I would worry that if the insurance company got a hold of Fitbit records out of context that they could –I could wind up with a higher premium or something like that.* *That’s just sensitive private information. Whether I have a cold or whether I have a more serious ailment disease, I think that’s private. Unless I wanna share it with people, that shouldn’t be disclosed to anyone without my expressed consent.*
Consumer attitudes	*I really don’t have nothing to hide, but that’s pretty personal to a lot of people, and I really wouldn’t like it. So I would just –I mean, you’ve got to have a little bit of freedom here. That’s what this country’s about.* *I think the worst ones would be my health bills. If that was not kept private, that would be really problematic. If you think about cell phone bills, electricity bills, internet bills, I don’t think I would have as much as an issue.*

#### Lack of Consumer Understanding of Digital Data Practices

Few participants could express a basic understanding of digital data collection practices, including how information is routinely tracked, stored, and subsequently used by third parties. Some were unaware that by downloading and using an app (eg, Facebook), they had consented to data collection. Participants who were aware that their data were being collected generally assumed the data were being used only by the entity collecting it and were not shared with third parties. Almost none of the participants named ways in which data are aggregated across sources and over time, a common practice by marketers to build profiles of individual consumers.

Participants generally did not recognize the connections between their digital data and inferences about their health. They were not able to conceptualize health information outside of a traditional definition of health care data. They were unaware that predictions or inferences about their health status could be drawn from sources like internet browsing information or social media posts.

#### Perceived Benefits and Harms of Digital Data Practices

Participants noted benefits of digital data collection to consumers and companies. They described the benefits of advertising highly tailored to their interests. They discussed convenience derived from data tracking. They discussed health benefits when prompted by specific health-related scenarios, but rarely mentioned them during other parts of the interview despite the interviewers introducing the study focus as related to health.

Indeed, most privacy concerns were broad rather than specific to health. Participants worried that their information could affect their finances, particularly that insurance coverage could be lost or premiums raised because of health information being made available to their insurer. Some participants feared losing employment opportunities if their information, especially health information, was available to an employer.

For some participants, the main harm was the intrusiveness of the digital data collection practices of the government, commercial companies like health insurers, and even health care providers, with access to information that some felt should remain private.

Participants split into two points of view when weighing tradeoffs between the benefits and risks of digital data collection and use. Some felt that it was a choice to give up privacy for convenience or other benefits. Several of these participants discussed tradeoffs in transactional terms. The use of discount cards, apps, and other programs naturally involves relinquishing some privacy, which is sometimes a worthwhile choice. Others described data tracking and sharing as inescapable. These participants did not consider these privacy risks acceptable but struggled to articulate how to avoid them.

#### Attitudes Regarding Digital Privacy

Despite a lack of knowledge on data privacy issues, participants strongly endorsed the significance of data privacy, based partly on a right to privacy and on concerns about harms.

When asked about privacy preferences for different types of data (health information, internet searches, and financial information), participants consistently expressed the greatest concern if their health information was not kept private. However, participants did not understand that data generated outside of health care could also be used to make inferences about their health.

### Part 2: Health-Related Applications of Consumer Digital Data

To make health applications of digital data more salient to participants, we prompted them with a series of hypothetical scenarios (Table 1) representing various sources, uses, and users of digital information for health purposes. For each scenario, participants were asked to provide their overall impression, describe what they liked and disliked about it, identify limits or protections they desired, and share whether they would want to participate.

Although participants’ responses to each scenario varied, certain themes were consistent. Participants appreciated the health benefits that could be achieved through the programs, including interventions to improve individual or population health, research to make advances in health care, and efforts to reduce health care costs. However, participants often expressed concern that many uses seemed too invasive and that their data might be used for other purposes beyond what was intended. They often desired protections so that data could be used only for the originally intended purpose, data could be kept private and secure, and participation could be voluntary and require consent. Table 4 provides illustrative quotes for the benefits, risks, and protections discussed by participants for each scenario.

**Table 4:** Consumer perspectives on digital data use scenarios (illustrative quotes).

Scenario^a^ (% favorable)	Rated favorable (N=45), n (%)	Benefit quotes	Risk quotes	Desired protection quotes
*A health insurance company accesses data on consumers’ grocery store purchases to identify individuals at high risk for developing diabetes and sends them healthy eating tips.*	20 (44%)	*I think it could give some valuable information. And especially if someone is unaware that their purchases are causing problems.* *The idea that someone is going to help me because I might not have that information that this product might not be good for my health, that is wonderful.*	*How much do you want them to stick their nose into what you’re doing? … Now they want to tell you what to eat? They could easily raise the premium on it. I know they would.* *That’s just my personal choice if that’s what I wanna purchase and I don’t need my health insurance charging me...*	*It’s just I don’t want them using it for different purposes, other than what … they were gonna use it for.* *I don’t think they should be able to use your –that information against you as far as, again, raising premiums or co-pays or deciding what’s a covered cost and what’s not a covered cost based on my lifestyle as far as what I eat.*
*A doctor’s office monitors internet search data of their patients and contacts individuals who search for symptoms of illness in order to provide an early diagnosis.*	12 (27%)	…*The doctor can be like hey, I seen you looked this up about measles and now all of a sudden you think you have measles –it’s all in your head.* *They can say, oh, I see that you’re looking for information on a child that’s throwing up chronically. Let’s get him in here and check him out...*	*They shouldn’t have access to what I’m searching for online because I am a Web M.D. queen…I will Google the symptoms and see if it’s something else or what I can do before I go to the doctor.* *It’s just an invasion of privacy. Again, if you are volunteering that information, then you can volunteer whatever information you want.*	*It would have to be on an opt-in basis so that people are made aware of what’s gonna happen and they’re choosing to let that happen* *That should definitely be protected, because that’s sensitive.*
*University researchers access the smartphone location and medical records of patients to study risk factors for cancer.*	35 (78%)	*I mean, that idea is something worth being monitored for because it’s for a good cause. I mean, it can potentially save hundreds of lives, and it can also save insurance companies.*	*Just I don’t like anyone knowing my whereabouts all hours of the day and all night, especially – and it’s not even just anyone. It’s – a stranger…* *Personally, no, because I don’t want them thinking because of where I work at that I’m buying tobacco all the time because I work at a store that sells tobacco and alcohol.*	*If there was a situation where I thought that someplace I went would give the wrong idea out of context, then just turn off GPS.* *Just knowing that they are not allowed to sell my information for anything else other than using it specifically for this research.*
*A technology company creates an app that tracks users’ locations and asks them to upload photos of their meals with the intention of encouraging healthy eating and sharing this information with advertisers who can provide users with coupons.*	27 (60%)	*I like that idea of if somebody wanted to do it so they don’t get diabetes. Upload any pictures of the food you eat and get tips about what to do.* *People have to actively want to use it and those individuals may want to receive grocery store coupons that are in line with the goals of losing weight, right? So I don’t have a negative reaction to that.*	*I would worry that in the future or – I think it’s already possible to – for them to sell the data to another company that could reverse engineer the data and result in higher insurance premiums for individuals and that sort of thing.* …*Nothing would prevent me from taking a picture of the salad that my wife is eating and post that as my dinner, while I’m – in reality, I’m eating a big, thick juicy steak and baked potato…It seems like a lot of effort to go through, and I’m not sure what the benefit’s going to be.*	*They have to disclose exactly who they’re sharing that information with outside of their company and the advertisers and what specific information they’re sharing with those advertisers and the other third parties that may be involved.* *I would think there would have to be a government mandate for a lot of transparency, including specific fines for misusing or not protecting the data.*

^a^Responses by participants to each scenario were coded as favorable, unfavorable, or neutral.

#### Scenario 1

Scenario 1 was a follows: *A health insurance company accesses data on consumers’ grocery store purchases to identify individuals at high risk for developing diabetes and sends them healthy eating tips.*

Overall, 20 out of 45 participants (44%) rated this program favorably. Participants liked the idea they could receive recommendations to improve their diet or management of their health conditions. Others thought the program could address population-level health problems in the United States or reduce insurance costs for those making healthy food choices. Participants were concerned by the motive of health insurers running such a program. They worried about losing insurance coverage or paying higher premiums based on their grocery purchases. Participants described protections they desired, including limits on other uses of data collected for this program, destruction of data after the program was complete, and opt-in and consent procedures for interested participants.

#### Scenario 2

Scenario 2 was as follows: *A doctor’s office monitors internet search data of their patients and contacts individuals who search for symptoms of illness in order to provide an early diagnosis.*

Overall, 12 out of 45 participants (27%) rated this program favorably. Participants identified several beneficial elements of this program, including the prevention of illness, possible savings on health care costs, convenience of care that could be provided remotely particularly for those with barriers to face-to-face care, and additional support from clinical providers for overall health. Additionally, participants liked that this scenario could provide patients with better quality information, preventing inaccurate self-diagnoses using web searches. Some participants felt that having a doctor access their searches would be too invasive, and they would rather conduct personal research and contact their provider if they had concerns. Some felt uncomfortable with the idea that their data could potentially be used for targeted drug or treatment advertising.

Participants emphasized the negative aspects of this scenario. Many were concerned about inadequate protections for their internet search data. They asked for full transparency and limits around the uses of their data, as well as personal data access. Participants also generally preferred that this program be opt-in and wanted assurances that there would be adequate data security safeguards in place.

#### Scenario 3

Scenario 3 was as follows: *University researchers access the smartphone location and medical records of patients to study risk factors for cancer.*

Overall, 35 out of 45 participants (78%) rated this program favorably. Participants saw benefit in participating in any program that could lead to cancer prevention, often citing personal or family history as motivators for participation. Some viewed this scenario more favorably because consent is required for research participation. Some participants who did not approve felt that the data sharing required was an invasion of their privacy. In particular, some participants indicated that they were not comfortable with researchers having access to their medical records.

Participants suggested that researchers obtain additional consent to have their location tracked and that they have the ability to turn off their location sharing at will. Participants also indicated the importance of transparency, specifically that the location information was secure, was not sold to other companies, and was used solely for research purposes. Finally, participants desired data to be deidentified.

#### Scenario 4

Scenario 4 was as follows: *A technology company creates an app that tracks users’ locations and asks them to upload photos of their meals with the intention of encouraging healthy eating and sharing this information with advertisers who can provide users with coupons.*

Overall, 27 out of 45 participants (60%) rated this program favorably. Those who responded favorably to this program thought it would help them to eat healthier and prevent diabetes, and many wanted to receive coupons for using the app. A few appreciated the voluntary or opt-in nature of the app. Others were mistrustful of the motives and felt that only the company or insurers would benefit.

Many participants did not want their information to be sold to advertising companies. Some would not participate because of the inconvenience of having to use the app consistently or the nuisance of receiving advertisements. Others had privacy concerns and worried about downstream use of their information for other purposes. For many, the only desired protection was the ability to either opt-in or opt-out of the app. Finally, participants discussed the importance of anonymity and the ability to use the app without advertisers or third parties identifying them.

## Discussion

### Principal Findings

In a broadly representative sample of US consumers, we examined attitudes and knowledge about digital health privacy. We identified several key findings. First, participants generally did not draw connections between the data they leave behind when they use digital technology and what it may reveal about their health. Second, participants struggled to weigh harms and benefits of data collection and use. Third, while preferences varied, participants generally wanted some protections in place for health-relevant consumer digital data.

Prior work has demonstrated that consumers value privacy but nonetheless continue to use digital technologies that routinely compromise that privacy [19]. Turow et al argued that this behavior should not be interpreted as consumers being unconcerned with privacy, but rather a sense of resignation that threats to privacy are unavoidable [20]. Our findings suggest that consumers often recognize that information is shared when they use digital technology but are often unaware of the extent of use or what can be learned about them from their data. Obar et al demonstrated that simply presenting consumers with more information will not necessarily lead to more informed consumers or yield higher quality decision making. In an experiment simulating registration with a social networking program, they found that consumers viewed lengthy privacy policies as a nuisance, felt overburdened by privacy statements, and missed “gotcha” provisions that were extreme (eg, many consented to give up their first-born child) [21].

Given that many consumers rank health privacy above other areas [22], it is concerning that most participants in our study could not draw connections between consumer digital data and inferences about their health. Broadly available analytic tools allow for the health status to be inferred from a wide range of data sources including smart devices in the home, language analysis from social media, financial spending habits, and internet search patterns [7,8,23]. Even when health applications were made explicit in the study scenarios, the tradeoffs between threats to privacy and benefits of use remained difficult for our study participants to reconcile. The difficulty assessing tradeoffs is likely compounded by the absence of limits on data use and the uncertain risks posed. Conger et al have argued that the relationships between consumers and businesses have become too complex for the individual consumer to assess given the involvement of third-party data brokers and open-ended uses of data [24].

Facing vignettes about commercial users, participants often raised concerns about economic harms (eg, higher insurance premiums and threats to employment) as well as unbridled secondary uses of their data. When considering vignettes with researchers and doctors as the data users, participants were often concerned with reputational harms (eg, sharing of embarrassing or unflattering information) [25]. Overall, participants responded much more favorably to some scenarios over others suggesting that consumers apply a nuanced evaluation of the use, including whether it is beneficial to themselves, the type of data that is being used, who is using their data, and the overall benefit of the use.

Participants sought privacy safeguards to reconcile the tradeoffs between health benefits and privacy. Many of the safeguards raised mirror those that have been part of the EU General Data Protection Regulation, including consent for data collection and use (opt-in), explicit statements of what data would be used for and by whom, limits on data transfer and selling, and data security requirements [26]. Participants were overwhelmingly in favor of protections for their digital health data. However, prior work points to practical limitations on the effectiveness of some of these approaches. For example, consumers often click through overly complex privacy agreements, suggesting a vastly simplified approach would be needed [20]. Policymakers might consider varying privacy protections based on the associated risks and benefits of different uses or curtail downstream transfers and aggregation of data that are far removed from the original point of collection.

### Limitations

This study has several limitations. Digital privacy is a topic with growing relevance and has been increasingly highlighted in popular media, which could shape consumers’ attitudes. Events just prior to interviews may have heavily influenced participants’ knowledge and perceptions. Therefore, the interviews reflect the time period (2018-2019) in which they were conducted. Despite efforts to recruit consumers with a wide range of characteristics, consumers who were willing to respond to and complete a request for an interview may have characteristics or attitudes not shared by those who chose not to participate. Social desirability bias may limit the credibility of participants’ expressed views. Despite these limitations, the interviews show a diverse array of attitudes regarding digital privacy.

### Conclusions

This study reveals how individual consumers wish to protect their own health information privacy and how little they are aware of the threats to that privacy from their conventional behaviors. Digital personal health information is now derived from consumer engagements as well as medical engagements. To the extent we need protections for personal health privacy, those protections need to extend beyond their current reach. Our study reinforces the finding that a purely market-based approach to privacy that depends on rational consumers making decisions with full information relies on faulty assumptions [27]. Future debates in the United States may need to focus on whether more robust privacy standards (similar to the European Union’s General Data Protection Regulation) are needed to protect consumer digital data from entities beyond health care systems [28,29].

## Appendix

Multimedia Appendix 1 Interview guide.
