# Supplementary material for: Consumer Views on Health Applications of Consumer Digital Data and Health Privacy Among US Adults: Qualitative Interview Study
Source: J Med Internet Res. 2021 Jun 9;23(6):e29395. doi: 10.2196/29395 (PMC8262668; doi:10.2196/29395)
Supplement: Multimedia Appendix 1 [file jmir_v23i6e29395_app1.docx]

**AIM 1: INTERVIEW GUIDE FOR CONSUMER INTERVIEWS**

*Introduction:*

Thank you for your interest in sharing your thoughts. I am part of a research team at the University of Pennsylvania. Our goal is to learn more about how people feel about digital technology.

Our conversation should last about 30-45 minutes. Please know that there are no right or wrong answers. We are interested in your thoughts and opinions.

Everything you say will remain confidential. I will be recording this session so that I can spend more time listening and talking with you rather than taking notes, but the recording will only be available to our research team, and will be destroyed after transcription. I’m not recording this right now, and I will only begin recording after you tell me it’s ok to begin. Does that sound alright to you?

Do I have your permission to begin the interview?

Many Americans use digital technology in the course of everyday life. Sometimes the information left behind can be used for different purposes. I’m interested in talking to you about how some of that information might be used for health-related reasons.

*Part 1:*

First, I want to ask you about how you use technology. Can you tell me about how you use technology in your day to day life?

Next, I’m going to ask you to take out your mobile phone if you have one. What apps or tools do you have on your phone? *[wait for list]* Which three are most important to you? I’d like to ask you some questions about those. *[wait for response]* Let’s talk about *[insert app here].*

- What is your understanding of what information this app collects about you?
- What is your understanding of what they might do with that information?
- What could someone else learn about your health if they had access to information about you from this app?
- What are some risks/ benefits of using this app? *(alternate order)*
  - *Prompt for more at least once*

*Part 2:*

Now I’m going to describe some different sources of digital information that could be used for different health or health care reasons. I will give a brief definition of each for clarity, even if the source may seem obvious to you. For each I want you to tell me on a scale from 0 to 100 how important it is that there are protections in place to keep the information private. 0 means that it is not important to have protections that keep the information private, and 100 means that it is extremely important to have protections that keep the information private.

- Electronic health record – your medical information or history that is collected by your doctor and stored on a computer
- Commercial genetic profile – the results about your DNA that come from a service where you send in a saliva sample to a company, such as 23andMe or Ancestry.com
- Electronic toll collection device – a device you put on your car windshield that pays tolls automatically when you drive through
- Fitbit or other wearable fitness tracker – a device you wear, for example on your wrist like a bracelet, that records your movement or activity
- Call log – the list of previous outgoing and incoming calls on your telephone
- Voicemail – the recordings people leave on your phone’s answering machine when you don’t pick up
- Texts – the electronic messages you send from your cell phone
- Photos (from cell phone) – pictures that are stored on your phone that you took with the phone camera or saved
- Social media posts – the record of things you have posted from social media accounts, such as a Facebook status, a tweet on Twitter, or a picture on Instagram
- Social media activity – things you do on social media besides post, such as like or favorite someone else’s post or interact with other content
- Emails – the record of your electronic correspondence
- Nest thermostat – a smart thermostat which is internet connected and allows you to control the temperature in your home
- Nest camera – a home security device where you can view the footage from your phone or computer
- Credit report – a number that represents your credit history based on your previous borrowing
- Credit card statement – a list of purchases paid for with a credit card in the past month
- Frequent flyer account – a loyalty program that has information on your travel history with a specific airline
- GPS navigation (from car) – the location information that is recorded when you look up your location or directions while driving
- Smart phone location – the records of locations you have visited that is collected from various apps, such as Google Maps
- Internet browser history – the record of your activity on your computer such as all the websites you have visited
- Grocery store rewards card – a card from a loyalty program with a grocery store that tracks you purchases at the store and can provide discounts
- Online reviews – reviews of a product or service that you post online, such as if you left a rating of a restaurant or product you bought

For the EHR, highest, middle, and lowest rated items:

*Tell me about why you chose [XX] for [source]. What about it made you feel like it was important or not important to keep private?*

*Part 3:*

For the third part of the interview, I’m going to describe some uses of consumer digital information and for each example ask you to talk about whether you think it is a good idea or a bad idea and why you think so. Let me start with the first example.

*Alternate scenario order, with scenario 1 always being 3^rd^ or 4^th^*

**Scenario 1**

A health insurance company is trying to find ways to keep people healthier and save money. They have found that consumers that buy certain kinds of food are more likely to develop diabetes. The insurance company is planning a program where they will access the grocery shopping records of their patients from grocery stores. The health insurance company will use this information to find out who is at high risk of developing diabetes, then send those people tips and advice on how they can prevent diabetes by making changes to the food they buy.

- *What do you think about this idea?*
- *Positive response 🡪 what things do you like? What would make you not like it?*
- *Negative response 🡪 what things don’t you like? What could make you like it?*
  - *If your first concern could be addressed, is there anything else that worries you?*
  - *If methods issues raised: What if methods issues could be addressed?*
- *Are there any limits or protections you think are necessary?*
- *If your insurance company offered a program like this, would you want to participate? Why/why not?*

**Scenario 2**

A doctor’s office is trying to find ways to prevent people from getting sick and needing to go to the hospital. They have found that patients that search on the internet for certain symptoms are more likely to get sick and need to go to an emergency room. This doctor’s office is planning a new program where they will access internet searches of their patients and contact patients that search for certain symptoms to try to start treatment sooner.

- *What do you think about this idea?*
- *Positive response 🡪 what things do you like? What would make you not like it?*
- *Negative response 🡪 what things don’t you like? What could make you like it?*
  - *If your first concern could be addressed, is there anything else that worries you?*
  - *If methods issues raised: What if methods issues could be addressed? (e.g. What if they could tell if you’re searching for yourself? What if searches did reveal serious illness?)*
- *Are there any limits or protections you think are necessary?*
- *If your doctor offered a program like this, would you want to participate? Why/why not?*

**Scenario 3**

University researchers are trying to find ways to prevent cancer. Researchers at a nearby university hospital are starting a research study where they will track patients over time to try to determine causes of cancer. In addition to using medical records, the research team will use location information from patient’s smartphones so they can study how the places where people spend most of their time impact their risk of getting cancer. The researchers want to use this knowledge to help develop public health strategies in the future that could reduce the number of people with cancer.

- *What do you think about this idea?*
- *Positive response 🡪 what things do you like? What would make you not like it?*
- *Negative response 🡪 what things don’t you like? What could make you like it?*
  - *If your first concern could be addressed, is there anything else that worries you?*
  - *If methods issues raised: What if methods issues could be addressed? (e.g. What if location information did provide insight into causes of cancer?)*
- *Are there any limits or protections you think are necessary?*
- *If a local university offered a program like this, would you want to participate? Why/why not?*

**Scenario 4**

DigiHealth is a company selling a new smartphone app that can automatically collect and store information on places users visit and the food they eat so that it can give advice on ways to lower their risk of obesity. The app tracks where users go using location services on their smartphone and tracks what they eat by having them upload a picture of their meals. DigiHealth is able to offer the App for free because it shares user information with advertisers so they can send out grocery coupons.

- *What do you think about this idea?*
- *Positive response 🡪 what things do you like? What would make you not like it?*
- *Negative response 🡪 what things don’t you like? What could make you like it?*
  - *If your first concern could be addressed, is there anything else that worries you?*
  - *If methods issues raised: What if methods issues could be addressed?*
- *Are there any limits or protections you think are necessary?*
- *If DigiHealth offered an App like this, would you want it?*

*Part 4:*

For the last part of the interview, I have just a few more questions. Can you tell me about any situations you’re aware of in which someone’s personal information was not kept private when it should have been?

Any situations involving you or someone you know?

What do you think about that?

Next, I’m going to ask you about three different types of information not being kept private.

1. How would you feel about you internet searches not being kept private?
2. How would you feel about your bills not being kept private?
3. How would you feel about your health information not being kept private?

Thinking about all three together, can you compare your concerns about each type of information not being kept private?

For the last question, can you tell me about how you use technology at work specifically?
